# Characterization of Patients with Chronic Myeloid Leukemia Unresponsive to Tyrosine Kinase Inhibitors Who Underwent Allogeneic Hematopoietic Stem Cell Transplantation

**Published:** 2017-01-01

**Authors:** Franceli Ramos Carvalho, Joice Zuckermann, Alessandra Paz, Gustavo Fischer, Liane Esteves Daudt, Lisandra Della Costa Rigoni, Lúcia Silla, Laura Fogliatto, Simone Martins de Castro, Diogo André Pilger

**Affiliations:** 1 Department of Analyses, School of Pharmacy, Universidade Federal do Rio Grande do Sul (UFRGS), Porto Alegre, RS, Brazil; 2 Service of Pharmacy, Hospital de Clínicas de Porto Alegre (HCPA), Porto Alegre, RS, Brazil; 3 Service of Hematology and Bone Marrow Transplantation (HCPA), Porto Alegre, RS, Brazil

**Keywords:** Chronic myeloid leukemia, Tyrosine kinase inhibitors, Resistance, Intolerance

## Abstract

**Background:** Tyrosine kinase inhibitors (TKIs) were the first drugs to use an intracellular signaling molecule as a therapeutic target. Unresponsiveness to TKIs limits therapeutic options, making allogeneic hematopoietic stem cell transplantation (HSCT) the only option leading to molecular remission. The aim of this study is to characterize CML patients unresponsive to first- and/or second-generation TKI therapy who underwent HSCT and to describe the main factors associated with treatment failure.

**Subjects and Methods:** Twenty one CML patients who underwent allogeneic HSCT and had previously used first- and/or second-generation TKIs from January 2005 to May 2014.

**Results:** Of the 21 patients, 52.4% were male, with a median age of 49 years (23-65 years) and 85.7% had chronic phase CML at the time of diagnosis; 28.6% showed inadequate treatment adherence to TKI therapy. Thirteen patients were resistant and eight were intolerant to TKIs; additionally, nine did not have T315I mutation. Ten transplantations involved related donors, and more than a half of patients (11) died, three of which due to graft failure. Most patients who survived transplantation were in the chronic phase of disease at the time of HSCT.

**Conclusion:** The population was composed mainly of young age patients at diagnosis, male, white, and coming from areas in the state of Rio Grande do Sul other than Porto Alegre and metropolitan region. Low adherence to TKI therapy may be related to unresponsiveness to treatment, especially in patients with acquired resistance, or this low adherence, together with the presence of molecular changes, may have led to the need for HSCT.

## Introduction

 Chronic myeloid leukemia (CML) is a myeloproliferative disease characterized by the presence of Philadelphia (Ph) chromosome resulting from t(9;22) (q34;q11) translocation between the ABL oncogene on chromosome 9 and the BCL gene on chromosome 22. This chromosome abnormality leads to the production of a hybrid protein with increased tyrosine kinase activity, which causes the proliferation of tumor cells. There are several treatments available for CML, such as hydroxyurea, interferon-α and tyrosine kinase inhibitors (TKIs), as well as allogeneic hematopoietic stem cell transplantation (HSCT), the only curative treatment for this disease so far.^   1 ^ TKIs were the first treatment for CML to use an intracellular signaling molecule as a therapeutic target. Basically, these inhibitors aim to reestablish normal cell proliferation.^   2 ^ Imatinib mesylate (Glivec®; Novartis, Basel, Switzerland), the first TKI approved by the Food and Drug Administration (FDA) in 2001, has been found to induce hematologic remission in 99% of the patients and cytogenetic remission in 74% after 12 months of treatment, and is currently the first line therapy for CML in Brazil particularly at the public health system.^   1 ^ This drug also inhibits tyrosine kinase receptors for platelet-derived growth factor (PDGF), stem cell factor (SCF) and proto-oncogene c-Kit (CD117), as well as cellular events mediated by PDGF and SCF but does not inhibit other tyrosine kinases such as those present in BCR-ABL T315I mutation and in Src family.^   3 ^

Dasatinib (Sprycel®, Bristol-Myers Squibb, Princeton, NJ, USA) and nilotinib (Tasigna®, Novartis, Basel, Switzerland) are second-generation TKIs developed to benefit patients who did not achieve appropriate hematologic, cytogenetic and molecular remission with imatinib, especially due to the presence of additional molecular abnormalities, BCR-ABL overexpression, or drug elimination by leukemic cells.^   4 ^ However, TKIs induce cytogenetic responses in most patients, onset of resistance, intolerance or lack of adherence to treatment are recognized as an important problem in the treatment of CML.^   5 ^

The use of first- and second-generation TKIs before HSCT allows for patients with accelerated or blast phase CML, who previously would not have survived to benefit from transplantation, to become candidates for the procedure.^   6 ^ Allogeneic HSCT is still the only curative treatment for CML; however, the morbidity and mortality treatment related after transplantation and development of graft-versus-host disease (GVHD) are great challenges when performing this procedure.   ^7^^,^^8^ 

The aim of the present study was to characterize a cohort of CML patients, treated in our institution, submitted to allogeneic HSCT due to unresponsiveness to first- and/or second-generation TKI therapy and describe the main reasons for treatment failure.

## SUBJECTS AND METHODS

 A retrospective descriptive study was conducted to analyze the medical records of 21 patients with any phase of CML (chronic, accelerated or blast) who underwent allogeneic HSCT at Hospital de Clínicas de Porto Alegre, a public university hospital in Porto Alegre, Rio Grande do Sul, Brazil, from January 2005 to May 2014. The study sample included male and female patients above 18 years of age and excluded patients who had not previously been exposed to TKIs. Sociodemographic variables included in the analysis were age, gender, skin color, region of origin and educational level. The clinical variables assessed were age at disease diagnosis, phase of disease at diagnosis, type of resistance (intrinsic/acquired) or intolerance (hematologic/non-hematologic toxicity) to treatment, presence of T315I mutation and treatment adherence. HSCT-related variables were also analyzed including phase of disease at transplantation, type of donor (related/unrelated), transplant outcome (survival/death), cause of death, time from transplantation to death and time from transplantation to data collection. Primary (intrinsic) resistance was defined as a lack of treatment efficacy from the onset of TKI therapy, and secondary (acquired) resistance was defined as an initial response followed by a loss of efficacy over time.^   9^ Treatment adherence was recorded by physician or the pharmaceutical team when dispensing drugs.

Data were analyzed using descriptive measures such as absolute and relative frequency of patients or median and interquartile range. This study was approved by Research Ethics Committee at Hospital de Clínicas de Porto Alegre (No: 140115).

## Results

 The study population consisted of 21 patients, most of whom were male (52.4%), white (90.5%), and came from areas in state of Rio Grande do Sul other than Porto Alegre and metropolitan region (57.2%). Median age was 49 years (23-65 years). The majority of the individuals had not completed primary education (38.1%). The most frequent age group at the diagnosis of CML was 21-40 years (61.9%) and most patients (85.7%) had chronic phase CML at diagnosis (Table 1).

**Table 1:** Sociodemographic and clinical characteristics of 21 CML patients previously treated with TKIs who underwent allogeneic HSCT

**Variable**	**N (%)**
Gender	
Female	10 (47.6)
Male Skin color White Non-white	11 (52.4) 19 (90.5) 2 (9.5)
Region of origin	
Porto Alegre and metropolitan region	7 (33.3)
Other area in the state of Rio Grande do Sul Other Brazilian state or other country	12 (57.2) 2 (9.5)
Educational level	
Incomplete primary education Complete primary education Incomplete high school education Complete high school education	8 (38.1) 5 (23.8) 3 (14.3) 4 (19.0)
Incomplete higher education Age at diagnosis < 21 years 21-40 years 41-60 years Phase of CML at diagnosis Chronic phase Accelerated phase Blast phase	1 (4.8) 1 (4.8) 13 (61.9) 7 (33.3) 18 (85.7) 2 (9.5) 1 (4.8)

**CML:** Chronic myeloid leukemia

**Figure 1:**
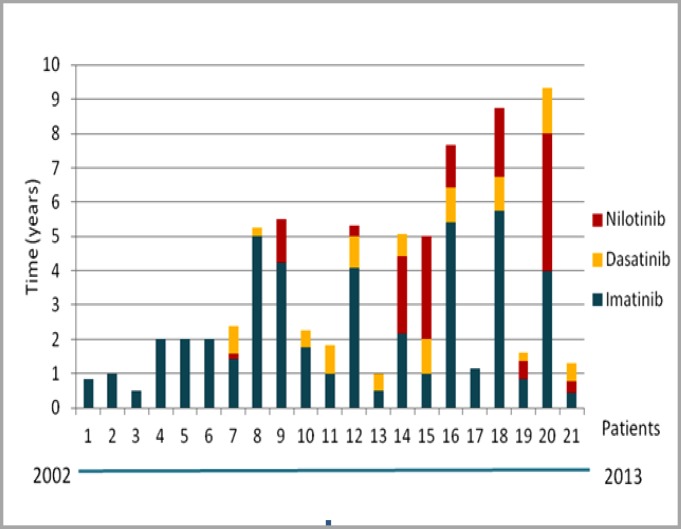
Duration of first- (imatinib) and second-generation (dasatinib and nilotinib) therapy per patient before HSCT. Patients are identified numerically, respecting the chronological order in which treatment started from 2002 to 2013, with patient 1 being the first and patient 21 last patient

**Figure 2:**
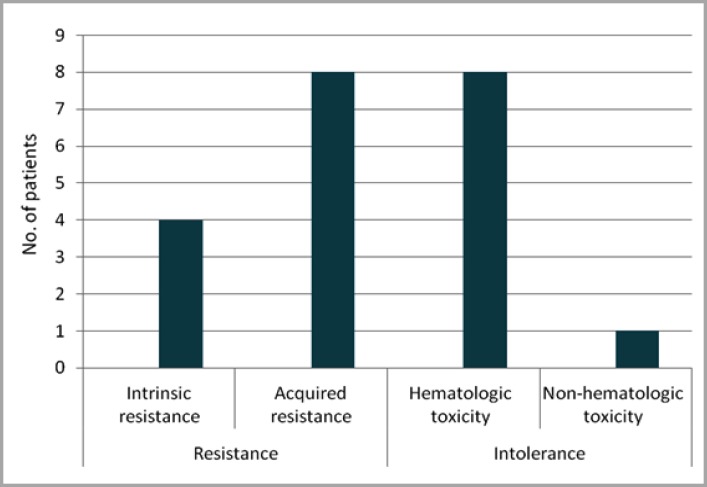
Distribution of types of resistance and intolerance in patients treated with first- and/or second-generation TKIs

Treatment adherence and treatment resistance due to presence of T315I mutation were also assessed. Thirteen (61.9%) patients showed adequate treatment adherence to first- and second-generation TKI therapy, six (28.6%) showed poor treatment adherence and two (9.5%) had missing information for this topic in their medical records. With regard to mutation analysis, one (4.8%) patient had a T315I mutation. Eleven (52.4%) patients were not investigated for mutation or this information was not available in their medical records. However, it is important to note that some of these patients were diagnosed with CML before mutation analysis was available, which explains the increased number of patients for whom this information was not available (Table 2). Duration of TKI therapy per patient showed that the minimum duration of TKI therapy before HSCT was 6 months (patient 3), and maximum duration was 9 years and 4 months (patient 20), showing that there was a great variability in the duration of TKI therapy (Figure 1). Seven patients received first-generation TKIs (patients 1, 2, 3, 4, 5, 6 and 17) and did not receive any second-generation TKI subsequently. The first six patients used only imatinib because it was the sole TKI available at the time of their treatment. Patient 17 was the only carrier of a T315I mutation. Moreover, there was no standardization on which second-generation TKI therapy to start after discontinuing imatinib, since some patients were given dasatinib and others, nilotinib. This may be mainly explained by which drug was available at the time of treatment or by patient's clinical conditions. The main reasons for replacing first-generation TKI therapy with second-generation TKI therapy and for deciding to undergo HSCT (in case of the seven patients who used only the first-generation TKI) were resistance (12 patients) or intolerance to TKIs (9 patients). Four patients with TKI-resistant CML had intrinsic resistance, whereas eight patients had acquired resistance. Nine patients were intolerant to TKI therapy, eight of which developed hematologic toxicity and one developed non-hematologic toxicity (hepatotoxicity) (Figure 2). As for the phase of CML at the time of HSCT, 11 patients were in the chronic, six in the accelerated and four in the blast phase. Of the 21 HSCTs performed, 10 involved related donors, all of which were patients' siblings. More than a half of patients (11) died, nine within the first 6 months after HSCT.

**Table 2:** Treatment adherence and presence of mutation in 21 CML patients who used TKIs

**Factor**	**N (%)**
Treatment adherence	
Yes	13 (61.9)
No	6 (28.6)
Not reported	2 (9.5)
Investigation for T315I mutation	
Absence Presence Not performed or not reported	9 (42.8) 1 (4.8) 11 (52.4)

**Figure 3:**
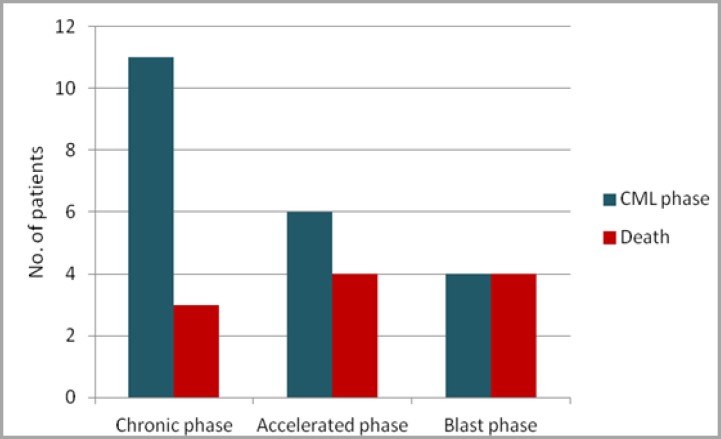
Association between mortality and phase of CML at transplantation

Three of 11 patients died due to graft failure and eight due to other complications such as stroke (one), chronic renal failure (two), GVHD (one), respiratory failure (one), post-transplantation relapse (two) and sepsis (one). With regard to the patients who survived HSCT (10), time from HSCT to data collection was less than one year in three patients, from 1-5 years in four patients and from 5-10 years in three patients (Table 3). Based on analysis of association between post-HSCT mortality and phase of CML, mortality was found to be more frequent in patients with blast phase CML (4/4) and accelerated phase CML (4/6) and less frequent in those with chronic phase CML (3/11) (Figure 3). 

**Table 3:** Allogeneic HSCT in 21 CML patients unresponsive to TKIs

**Variables**	**N (%)**
Phase of CML at the time of HSCT Chronic phase Accelerated phase Blast phase	11 (52.4) 6 (28.6) 4 (19.0)
Allogeneic HSCT Related donor Unrelated donor Death 0-6 months after HSCT 7-12 months after HSCT Causes of death Graft failure Other complications Patients who survived HSCT 0-1 year after HSCT 1-5 years after HSCT 5-10 years after HSCT	10 (47.6) 11 (52.4) 9 (42.8) 2 (9.5) 3 (14.3) 8 (38.1) 3 (14.3) 4 (19.0) 3 (14.3)

**Figure 4:**
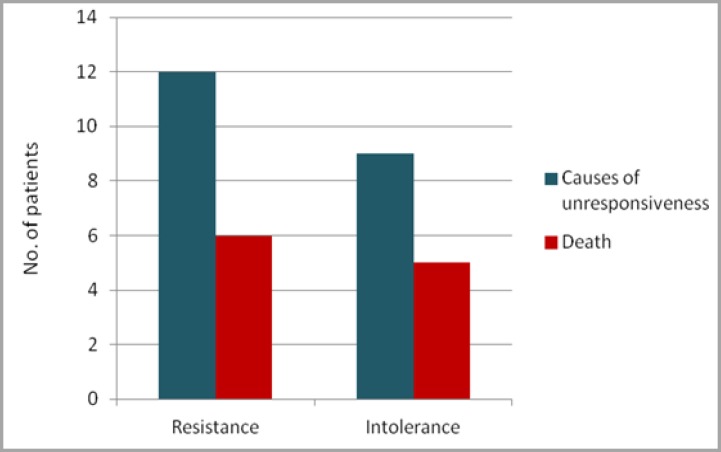
Association between mortality and presence of resistance or intolerance

The association between mortality and causes of unresponsiveness to TKI therapy (resistance and intolerance) was also assessed. This analysis yielded similar results between intolerant and resistant patients, since half (six) of twelve patients identified as treatment resistant died, as well as five of the nine treatment-intolerant patients (Figure 4).

## Discussion

 This study allowed, for the first time, to characterize patients who underwent HSCT and used first- or second-generation TKIs and to determine reasons for therapeutic failure, especially considering data on treatment adherence and resistance. With regard to adherence, more than 30% of the patients not showed adequate adherence to first- and/or second-generation TKI therapy, which may have influenced unresponsiveness to TKI therapy. A study with 87 patients with chronic phase CML treated with imatinib 400 mg daily found that major molecular response was achieved in only 28.4% of patients with adherence rates of 90% or lower vs. 94.5% of those with adherence rates greater than 90%. Complete molecular response rates were 0% vs. 43.8%, respectively. No molecular responses were observed when adherence rates were 80% or lower.  ^10^^,^^11^ Low adherence may also result in treatment failure because resistant clones may emerge more easily.

Additionally, illiteracy and low cognitive levels are important factors that may hamper patients' understanding of treatment instructions.  ^12^^,^^13^ In clinical practice, pharmaceutical care is commonly used as a tool to improve clinical outcomes of drug therapy, with purpose of identifying, preventing and solving drug-related problems and educate patients on the importance of treatment adherence. Our results suggest that low adherence to TKI therapy may be related to unresponsiveness to treatment, especially in patients with acquired resistance or that low adherence, together with presence of molecular changes may have led to need for HSCT. Patients with poor treatment adherence reported forgetting to take medications, depression characteristics leading to treatment dropout or presence of therapy-related adverse effects such as vomiting, diarrhea, abdominal pain or dermatological problems. The present study found T315I mutation in 4.8% of patients. However, a significant portion of patients (52.4%) had missing data on mutation analysis, a limitation of this study that makes it difficult to establish a relationship between the presence of mutation and resistance to TKIs. Treatment resistance was observed in 12 patients, with a predominance of acquired resistance and intolerance to TKIs was present in nine patients who did not respond to treatment. Normally, these patients develop drug toxicity and are not able to take it at the recommended dose to achieve treatment response. In eight patients, drug intolerance resulted from hematologic toxicity, with findings such as thrombocytopenia, leukopenia or anemia.

A minority of patients with chronic phase CML and a substantial proportion of those with accelerated and blast phase CML are either initially refractory to imatinib or lose imatinib sensitivity over time or cannot tolerate treatment side effects (toxicity). Resistance to imatinib therapy is observed in nearly 10-15% of patients and may be classified as primary or secondary, according to the time of onset.^12^ Point mutations are one of the most common mechanisms of resistance to TKI therapy. Second-generation TKIs are able to overcome most mutations that cause resistance to imatinib, although new mutations that make leukemia resistant to dasatinib and/or nilotinib. One of these mutations is T315I mutation, which has shown to be resistant to all TKIs currently available in Brazil. Mutations in BCR-ABL gene are described in 42-90% of patients resistant to TKIs. Patients resistant to imatinib are more likely to develop additional mutations associated with resistance to second-generation TKIs, thus making HSCT an inevitable procedure.  ^6^^,^^14^ An analysis of medications used by each patient included in present study revealed that minority of patients received only one TKI (imatinib) because it was only available in hospital at the time of their treatment. Although use of imatinib as first therapeutic strategy has drastically changed outcome of CML patients, one third of these patients do not achieve optimal outcomes and require alternatives therapies due to the onset of resistance or intolerance. Eight-year follow-up of international IRIS study showed 85% overall survival rate but 30% of patients had unfavorable outcome, mostly due to primary (17%) or acquired resistance (15%).^   15^ Duration of TKI therapy per patient was considerably variable (6 months to 9 years), which may also be explained by peculiarities of each treatment. In present study, patients with longer treatment time were more likely to develop acquired resistance i.e. they had adequate response for some time and then lost response during treatment. Some patients were initially opposed to decision of undergoing HSCT and prolonged use of TKIs, while others had difficulty to find a compatible donor because they did not have a compatible related donor.

The emergence of dasatinib and nilotinib provided patients with a second treatment option with greater potential and specificity than imatinib.^   16^ Patients who started taking second-generation TKIs after imatinib failure may experience a secondary failure. Therefore, response to next-generation TKIs should be carefully monitored and assessed as early as possible.^   17^ Even after use of TKIs, allogeneic HSCT plays an important role in treatment of CML patients due to the following reasons: (1) up to one third of patients with first chronic phase CML are resistant or intolerant to imatinib therapy, (2) patients with T315I mutation are highly resistant to all TKIs, (3) young patients with identical related donor have high rates of cure, (4) patients with advanced disease have poor response to TKIs and (5) outcomes of HSCT have improved in last few years.^   7^

HSCT was historically used in initial treatment of young patients newly diagnosed with CML who had an appropriate donor but currently is almost reserved to patients resistant or intolerant to TKIs and to rare cases when TKIs are not available.  ^6^^,^^14^^,^^18^ In IRIS trials, 15-25% of patients experienced failure with imatinib therapy due to intolerance or resistance to treatment, which occurred at an annual rate of 4%.^   7^ Allogeneic HSCT is still only curative modality for CML and its use in carefully selected patients has been increasingly recognized. In present study, we observed that four and six patients were in blast and accelerated phase of disease, respectively, at time of HSCT.  ^7^^,^^19^ However, previous studies have demonstrated that clinical outcomes of allogeneic HSCT in CML depend on pre-transplant parameters. Similarly, previous studies have established that allogeneic HSCT, when performed in early chronic phase of disease, is associated with improved patient survival. ^   6^ Thus, it was shown that patients with chronic phase CML at the time of allogeneic HSCT had better survival rates than patients with accelerated and blast phase CML at transplantation. The association between post-HSCT mortality and presence of resistance or intolerance was similar. In both cases, CML may progress to accelerated or blast phases, which may considerably reduce patient's survival, as previously shown. Thus, there is a need to assess impact of pre-transplant therapies, especially those with nilotinib and dasatinib, since this is a recent approach. In this context, it is particularly interesting to determine whether HSCT should be the second or the third strategy in case of imatinib failure in CML patients. Finally, role and duration of TKI therapy in this scenario also warrant further investigation.  ^19^^,^^20^

## CONCLUSION

 The present study allowed us to characterize patients who underwent allogeneic HSCT after showing resistance or intolerance to treatment with first- and/or second-generation TKIs. The study population was composed mainly of patients of young age at diagnosis, male, white and coming from areas in state of Rio Grande do Sul other than Porto Alegre and metropolitan region.

It is worth highlighting the importance of molecular monitoring on assessment of association between unresponsiveness to TKI therapy and treatment resistance, which contributes to future decision to undergo HSCT. With this study, it was possible to observe that several factors such as treatment adherence and presence of drug resistance or intolerance, contribute to unresponsiveness to TKI therapy.
